# Crosstalk between gut microbiota and renal ischemia/reperfusion injury

**DOI:** 10.3389/fcimb.2022.1015825

**Published:** 2022-09-05

**Authors:** Peng Huang, Jianwei Cao, Jingyi Chen, Yanrong Luo, Xiaofang Gong, Chengyi Wu, Yu Wang

**Affiliations:** ^1^ Department of Anesthesiology, Taihe Hospital, Hubei University of Medicine, Shiyan, China; ^2^ Department of Microscopic Orthopedics of Hand and Foot, Taihe Hospital, Hubei University of Medicine, Shiyan, China; ^3^ Physical examination center, Shiyan Hospital of Integrated Traditional and Western Medicine, Shiyan, China

**Keywords:** intestinal microbiota, metabolites, renal ischemia reperfusion, intestinal dysbiosis, biomarkers

## Abstract

Renal ischemia-reperfusion injury (IRI) is the main cause of acute kidney injury and the cause of rapid renal dysfunction and high mortality. In recent years, with the gradual deepening of the understanding of the intestinal flora, exploring renal IRI from the perspective of the intestinal flora has become a research hotspot. It is well known that the intestinal flora plays an important role in maintaining human health, and dysbiosis is the change in the composition and function of the intestinal tract, which in turn causes intestinal barrier dysfunction. Studies have shown that there are significant differences in the composition of intestinal flora before and after renal IRI, and this difference is closely related to the occurrence and development of renal IRI and affects prognosis. In addition, toxins produced by dysregulated gut microbes enter the bloodstream, which in turn exacerbates kidney damage. This article reviews the research progress of intestinal flora and renal IRI, in order to provide new treatment ideas and strategies for renal IRI.

## Overview

Renal ischemia-reperfusion injury (IRI) is the main cause of acute kidney injury and the cause of rapid renal dysfunction and high mortality (Zhang et al., 2020; Ke et al., 2021; Zhang et al., 2021a; Zhou et al., 2021). It is a complex but not fully understood process. In recent years, more and more studies have found that intestinal flora is associated with renal IRI (Ding et al., 2019). There are millions of microorganisms living in the human body (Liu et al., 2021; Lu et al., 2021; Wang et al., 2021). These microorganisms are widely present in the skin (Boxberger et al., 2021), gastrointestinal tract (Chen et al., 2021), nasal cavity (Bassis et al., 2014), oral cavity (Blaustein et al., 2021) and reproductive tract. In a healthy organism, they cooperate and influence each other in ways that do not cause disease. And multiple studies have found a strong link between gut microbiota and kidney disease (Hu et al., 2017; Ticinesi et al., 2018; Gryp et al., 2020). This article reviews the research progress of gut microbiota and renal IRI, in order to provide new treatment ideas and strategies for renal IRI.

## Renal IRI

Ischemia-reperfusion injury (IRI) refers to the phenomenon that when tissues or organs regain blood supply after ischemia, they do not recover the function of tissues and organs, but aggravate the dysfunction of function and metabolism and structural destruction (Deng et al., 2021a; Deng et al., 2021b; Deng et al., 2022a). Because of its special structure and function, kidney is also extremely sensitive to IRI. During renal transplantation, the transplanted kidney disorder inevitably experiences donor hemodynamic kidney disorder. IRI of the transplanted kidney can cause delayed recovery of graft function, primary nonfunction of the transplanted kidney, acute rejection, etc., which is an important factor affecting early renal function recovery and long-term survival of the transplanted kidney (Wu et al., 2021b; Feng et al., 2022; Kitani et al., 2022). Therefore, the prevention and treatment of renal transplantation IRI has become a hot topic in the field of renal transplantation in recent years.

## Intestinal microbiota

Intestinal microbiota plays a very important role in a series of pathophysiological processes such as resistance to pathogen invasion, establishment of body immunity, nutrient digestion and absorption, body growth and metabolism, immune anti-tumor and so on (Daoust et al., 2021; Delaroque et al., 2021; Tsou et al., 2021; Zhao et al., 2021). It can interact with intestinal epithelial cells, other organs and the whole body. There are a large number of immune cells and many immune factors in intestinal tissue, which play an important role in intestinal mucosal immunity. The gut is an important organ in the body, responsible for the digestion and absorption of nutrients. In addition, as the largest lymphoid organization inside the body, intestinal tract still holds the function that endocrine and immune regulate concurrently. Intestinal epithelial cells are the barrier between the body and the external environment. The microorganisms (intestinal flora) in the intestines are closely related to the metabolic and immune functions of the intestine. Studies have confirmed that the body’s homeostasis and the intestinal immune function depend on the balance of bacteria in different degrees, and once this balance is destroyed, it will lead to the occurrence of related diseases (Bruellman and Llorente, 2021; Han et al., 2021; Khan et al., 2021; Shute et al., 2021; Sun et al., 2021; Deng et al., 2022b; Hu et al., 2022). The dysregulation of gut microbiota can be caused by many factors, such as age (Du et al., 2021; Janiak et al., 2021), lifestyle (Pauer et al., 2021; Shin et al., 2021), dietary habits (Neumann et al., 2021; Tap et al., 2021; Yu et al., 2021), immunity (Foley et al., 2021; Hosseinkhani et al., 2021) and the use of antibiotics (Kang et al., 2021; Mcdonnell et al., 2021; Strati et al., 2021; Vicentini et al., 2021).

## Gut-kidney axis

In 2011, Professor Meijers first proposed the “gut-kidney axis” theory (Meijers and Evenepoel, 2011). The core of the theory is that chronic renal failure causes colonic microbiota disorder, and pathogenic bacteria produce metabolic toxins, mainly indoxol sulfate and p-cresol. These toxins enter the circulation and lead to systemic inflammation and oxidative stress injury, thus aggravating kidney injury. With the popularization of metagenomics and metabolomics technology in recent years, the relationship between “microbiota - toxin-barrier - inflammation” event chain has been clarified. The disruption of mucosal barrier caused by colonic microbiota disorder and the shift of bacterial metabolites are considered as the most core link of the whole “gut-kidney axis” (Yang et al., 2018; Giordano et al., 2021).

There are interactions between gut microbiota and various organs of human body, and there is a bidirectional synergistic relationship between gut and kidney (Wang et al., 2020; Wu et al., 2020; Lai et al., 2021; Lauriero et al., 2021; Xiang et al., 2021). Under normal physiological conditions, the intestine has a regulatory effect on kidney function, including neuroendocrine involvement. For example, glucagon-like peptide-1 (GLP-1) (Wu et al., 2021a; Zhuang et al., 2021), guanosine (Estaki et al., 2014), glucagon (Müller et al., 2020; Insuela et al., 2021), vasoactive intestinal peptide (Song et al., 2021), polypeptide (Qi et al., 2021), gastrin (Stewart et al., 2020) and other intestinal factors can regulate renal dysfunction. Pathological conditions, due to dietary restrictions and gastrointestinal, most of the patients in the gut microbial metabolism, which is mainly composed of protein hydrolysis into fermentation mode, the end product of protein by the metabolism of gut microbes such as phenol, indole are the endotoxin source of uremia, such as its nephrotoxicity and vascular toxicity has been confirmed that many clinical research institute (Ondrussek-Sekac et al., 2021). Intestinal metabolites of host and flora affect the whole body through the blood circulation, in addition to the intestinal nervous and immune systems, the brain, gut, flora and kidney are closely related and promote disease. On the other hand, renal IRI can destroy the composition and metabolism of intestinal microbiota, and long-term imbalance of intestinal microbiota can lead to renal injury, such as secondary IgA nephropathy induced by intestinal diseases (Yang et al., 2020). Any interference between this bidirectional communication can lead to a variety of serious complications, such as chronic kidney disease, end-stage renal disease, and septic acute kidney injury.

Not only that, intestinal tract plays an important role in regulating blood sodium and potassium metabolism (Olson et al., 2018). The absorption of sodium and potassium in the intestine changes the blood concentration and stimulates the renin-angiotensin system, which leads to the change of the excretion of sodium and potassium in the kidney to maintain the blood sodium and potassium concentration relatively normal. Long-term intestinal sodium and potassium absorption disorder makes the kidney to sodium and potassium concentration sensitivity retardation and glomerular filtration rate decrease, resulting in renal function damage. When intestinal damage loses the ability to regulate blood phosphorus, it will also increase the metabolic burden of the kidney.

## Gut microbiota and immunity

The intestinal tract is the largest immune organ. Intestinal microbiota and intestinal mucosal immune cells not only play a role in intestinal inflammatory diseases, but also control the immune response of extraintestinal organs. The over-activation of innate immune response plays an important role in the intestinal tract pathogenesis of acute kidney injury. Therefore, gut microbiota and intestinal mucosal immune cells may affect the prognosis of acute kidney injury. As the “second genome of human beings”, gut microbiota is involved in the regulation of many metabolic pathways, such as human gene expression, nutritional development, adaptive immunity and so on. Studies of chemokines associated with T-cell infiltration have found that gut microbiota can stimulate the production of immune chemokines, thereby facilitating the killing of tumor cells by T-cells. Studies on gut microbiota and host immunity have confirmed that gut microbiota is an important factor to stimulate the maturation of “mucosal immune system” and “systemic immune system”, and gut microbiota can act as signal molecules to regulate intestinal development, angiogenesis and lymphocyte development. In addition, more and more studies have shown that the gut microbiota also plays an extremely important role in protecting the host from pathogenic microorganisms. For example, goblet cells in the intestinal mucosa secrete a mucin, which has a compact inner mucus layer and is resistant to bacteria. The outer mucus layer can compete with pathogenic microorganisms to bind adhesin receptors of epithelial cells, thus achieving the purpose of inhibiting the adhesion and colonization of pathogenic microorganisms in the intestinal tract. Lactobacillus in the gut can promote mucin expression. Some genera of *Firmicutes* have the ability to penetrate the mucus layer, and they can stimulate intestinal epithelial cells to produce a large number of antimicrobial proteins, thereby limiting the contact between bacteria and intestinal epithelial cells, and thereby inhibiting the colonization of pathogenic microorganisms in the intestine. Therefore, the homeostasis of intestinal flora plays an indispensable role in regulating the development of host immune system and maintaining normal immune function of the body.

## Diet-gut microbiota-kidney

### Dietary fiber

Dietary fiber is a source of carbohydrates for the microbiota (Coker et al., 2021; Zuo et al., 2022). The composition of the colonic microbiota is significantly influenced by diet and its assimilation in the small intestine (Murga-Garrido et al., 2021). Dietary fiber escapes the digestive process of the small intestine and becomes the main source of carbohydrates for the colonic microbiota. Dietary fiber is fermented by colonic microbiota into short-chain fatty acid, which play an important role in maintaining intestinal epithelial integrity and energy homeostasis, and they repair hypoxic damage in renal epithelial cells by improving mitochondrial biogenesis (Andrade-Oliveira et al., 2015).

### Dietary protein

Dietary protein digested in the upper digestive tract is a nitrogen source for colonic microbiota (Westerterp-Plantenga et al., 2009). The fate of these proteins in the colon is largely determined by the availability of energy for the growth and development of the colonic microbiota, which is largely derived from carbohydrate fermentation. If the availability of carbohydrates is high, proteins and their intermediates are either assimilated into bacterial biomass or fermentated into p-cresol, indole, phenol and amines by Clostridium and bacteroides in the absence of carbohydrates. The protein fermentation products are further processed to form uremic toxins such as p-cresol sulfate and indoxol sulfate (Rysz et al., 2021). These toxins circulate in the blood due to their high affinity (non-covalent interaction) with albumin and are released by renal tubular secretion. If uremic retained solutes accumulate in the body, they increase the incidence of glomerulosclerosis and the progression of kidney disease. Therefore, their concentration in the blood can be used to measure the functional efficiency of the kidney. Indole phenol sulfate and other toxic effects of cresol sulfate including increased inflammation, endothelial dysfunction and vascular calcification, oxidative stress increase, erythropoiesis reduced, increased cell aging, thrombosis, atherosclerosis formation, left ventricular hypertrophy, insulin resistance, renal tubular interstitial fibrosis and renin - angiotensin aldosterone system activation (Vanholder et al., 2014).

### Dietary fat

Choline, carnitine and lecithin are the main components of dietary fat (Higarza et al., 2021). Mammals lack the enzymes needed to break the cyanide bonds of these fatty components. However, the colonic microbiota has trimethylamine lyase, which breaks cyanide bonds. The combined action of trimethylamine lyase in bile and liver enzymes (namely flavin-containing monooxygenases) results in the formation of trimethylamine N-oxide by carnitine and choline. According to a 2021 study published in the journal Science, high-fat diets impair mitochondrial function in colonic epithelial cells, increase intestinal oxygen and nitrate concentrations, promote the growth of *Escherichia coli* and the breakdown of choline, lead to increased levels of trimethylamine, and ultimately, the harmful circulating metabolite trimethylamine N-oxide. Trimethylamine N-oxide, like other uremic toxins, enters the body’s circulation and is released by the kidneys. Increased trimethylamine N-oxide is directly associated with the progression of chronic kidney disease (Liu et al., 2020). trimethylamine N-oxide concentrations can be up to 20 times higher in patients with end-stage renal disease compared to healthy controls. High trimethylamine N-oxide results in deleterious consequences such as increased platelet activity, thrombogenic potential, renal tubulointerstitial fibrosis, and the development of atherosclerosis.

## Gut microbiota in renal IRI

### Changes in gut microbes and metabolites induced by renal IRI

It was found that renal IRI resulted in increased serum levels of 32 acylcarnitines, which were correlated with creatinine and urea. Levels of three amino acids (tyrosine, tryptophan and proline) decreased. In addition, there was a significant association between intestinal bacterial abundance and metabolite levels. The levels of *Rothia* and *staphylococcus* were positively correlated with creatinine and urea levels, respectively (Andrianova et al., 2020).

### Antibiotics

Currently, the effect of antibiotic deletion of gut microbiota on renal IRI remains controversial. Diba Emal et al. found that microbiota depletion significantly attenuated renal injury, dysfunction, and remote organ damage, and maintained renal tubule integrity after renal IRI. Compared with control mice, mice with intestinal microbiota depletion expressed lower levels of F4/80 and chemokine receptors CX3CR1 and CCR2 in F4/80^+^ renal resident macrophages and bone marrow monocytes. In addition, bone marrow monocytes from mice depleted of gut microbiota exhibited reduced migration to CX3CL1 and CCL2 ligands compared with control bone marrow monocytes. In addition, fecal transplantation in antibiotic-treated mice showed that fecal transplantation from untreated mice abolished the protective effect of microbiota depletion on renal IRI (Emal et al., 2017). Therefore, inhibition of inflammatory response by targeting microbiome derived mediators may be a promising therapeutic approach for renal IRI. Yang et al. found that increased *Enterobacteriaceae* and decreased *Lactobacillus* and *ruminococcus* were found to be markers of dysbiosis caused by kidney IR and were associated with reduced levels of short-chain fatty acids, intestinal inflammation, and intestinal leakage. Depletion of the microbiota by oral antibiotics prevents kidney IRI. This nephroprotective effect was associated with reduced Th 17, Th 1 responses and expansion of regulatory T cells and M2 macrophages. The study demonstrates a unique bidirectional relationship between kidney and gut during acute kidney injury. Dysregulation of the gut microbiota, inflammation and leaky intestines are the consequences of acute kidney injury, but they are also important factors in determining the severity of post-acute kidney injury (Yang et al., 2020). Therefore, targeting the gut microbiota may provide a novel therapeutic strategy for acute kidney injury.

However, Osada Y et al. reported that microbiome depleted mice had significantly lower bacterial 16S rRNA expression, luminal concentrations of short-chain fatty acids and bile acids, and plasma glucose levels than vector-treated mice. In addition, antibiotic treatment significantly reduced renal glucose and pyruvate levels. The mRNA expression levels of glucose 6-phosphatase and phosphoenolpyruvate carboxykinase in renal cortex of antibiotic treated mice were significantly higher than those of vector treated mice. Antibiotic treatment caused more severe tubular injury after renal IR. The study confirms that microbiome depletion is associated with reduced levels of pyruvate in the kidney, which may be caused by activation of renal gluconeogenesis. Microbiome depletion can increase the susceptibility of the kidney to IR injury (Osada et al., 2021). Gut microbiota protects against tubular injury in a mouse model of renal IR. Acute kidney injury induced dysregulation of intestinal flora leads to changes in D-amino acid metabolism. In injured kidneys, the activity of D-amino acid oxidase is reduced. Oral administration of D-serine attenuated renal injury in B6 mice and D-serine depleted mice. D-serine inhibits hypoxia-induced renal tubular injury and promotes proliferation of renal tubular cells after hypoxia. Finally, circulating D-serine levels were significantly associated with decreased renal function in acute kidney injury patients (Nakade et al., 2018).

### Probiotics

Zeng et al. found that oral administration of the probiotic *Lactobacillus casei Zhang* corrected intestinal microbial dysregulation induced by bilateral renal IR, attenuated renal injury, and delayed its progression to chronic kidney disease in mice. *Lactobacillus casei Zhang* increased serum and kidney levels of short-chain fatty acids and nicotinamide, thereby reducing renal inflammation and damage to renal tubular epithelial cells (Zhu et al., 2021). These results suggest that oral administration of *Lactobacillus casei Zhang* is a potential therapy to attenuate renal injury and slow the progression of renal decline by altering SCFA and nicotinamide metabolism. Administration of *Bifidobacterium bifidum BGN4* significantly increased gut microbiota diversity and prevented the expansion of *Enterobacteriaceae* and *Bacteroidetes*, hallmarks of AKI-induced dysbiosis. In addition, administration of *Bifidobacterium bifidum BGN4* significantly reduced IRI-induced other changes in the colonic microenvironment, including effects on colonic epithelial cell permeability, apoptosis, and infiltration of neutrophils and pro-inflammatory macrophages (Yang et al., 2021). *Lactobacillus acidophilus ATCC 4356* attenuates renal IRI and improves gut microbial distribution through anti-oxidative stress and anti-inflammatory responses (Zhang et al., 2021b).

### Short-chain fatty acids

Short-chain fatty acids are fermentation end products produced by the gut microbiota and have anti-inflammatory and histone deacetylase inhibitory properties. Recently, it has been observed that treatment with the three main short-chain fatty acids (acetate, propionate, and butyrate) improved renal dysfunction caused by injury by modulating inflammatory processes. In addition, short-chain fatty acids ameliorated the effects of hypoxia on renal epithelial cells by improving mitochondrial biosynthesis. Notably, mice treated with acetogenic bacteria also had better results after acute kidney injury (Andrade-Oliveira et al., 2015). Butyrate treatment significantly enhanced renal function and structure, decreased serum creatinine levels, and reduced pathological damage to renal tissue. With the recovery of renal function after kidney transplantation, short-chain fatty acids increased and were negatively correlated with creatinine (Sun et al., 2022).

## Conclusion

Intestinal microbiota and its products are closely related to renal IRI, and external factors such as diet structure and drug application have a great influence on intestinal microbiota. Therefore, in the management process of patients with renal IRI, the influence and interaction on intestinal microbiota should be taken into account when reasonable treatment options are selected. In addition, a deeper understanding of the function and composition of the gut microbiota of patients can provide a focal point for the development of rational and individualized treatment. It will be the focus of future research to carry out more studies on the intestinal microbiome of renal IRI, improve the metagenomic sequencing of intestinal microbiota in recipients, explain the mechanism of intestinal microbiota assisting the body’s immunity and the relationship between kidney diseases and intestinal microbiota. In view of the beneficial effects of probiotics on renal diseases and the efficacy and safety of fecal microbiota transplantation, individualized application of probiotics and fecal microbiota transplantation may become a new treatment strategy for renal IRI.

## Data availability statements

The authors confirm that the data supporting the findings of this study are available within the article.

## Author contributions

WY, HP and CJY contributed equally to the writing of this manuscript; CJW, LYR, WCY, and GXF designed the illustrations. All authors contributed to the article and approved the submitted version.

## Funding

This work was supported by grants from Shiyan Taihe Hospital Scientific Research Supporting Fund (2019SCI013 to Jingyi Chen).

## Conflict of interest

The authors declare that the research was conducted in the absence of any commercial or financial relationships that could be construed as a potential conflict of interest.

## Publisher’s note

All claims expressed in this article are solely those of the authors and do not necessarily represent those of their affiliated organizations, or those of the publisher, the editors and the reviewers. Any product that may be evaluated in this article, or claim that may be made by its manufacturer, is not guaranteed or endorsed by the publisher.
